# Risk Factors for Postoperative Pulmonary Compromise in a Pediatric Population: A Retrospective Review of a Single Institution Cohort

**DOI:** 10.3390/children12101403

**Published:** 2025-10-17

**Authors:** Alison Robles, Mehul V. Raval, Chunyi Wu, Heather A. Ballard, Mitchell Phillips, Nicholas E. Burjek, Eric C. Cheon

**Affiliations:** 1 Department of Pediatric Anesthesiology, Ann & Robert H. Lurie Children’s Hospital of Chicago, Northwestern University, Chicago, IL 60611, USA; cwu@luriechildrens.org (C.W.); hballard@luriechildrens.org (H.A.B.); mitchell.phillips@nemours.org (M.P.); nburjek@luriechildrens.org (N.E.B.); echeon@luriechildrens.org (E.C.C.); 2 Division of Pediatric Surgery, Ann & Robert H. Lurie Children’s Hospital of Chicago, Feinberg School of Medicine, Northwestern University, Chicago, IL 60611, USA; mraval@luriechildrens.org

**Keywords:** pulmonary ventilation, pediatric anesthesia, postoperative complication, lung injury

## Abstract

Background/Objectives: Pediatric postoperative pulmonary complication is a major event associated with increased in-hospital morbidity and mortality. However, data is limited regarding the specific timing and spectrum of postoperative pulmonary complications in the pediatric population. Utilizing data in a cohort of high-risk patients aged ≤ 6 years, we sought to evaluate the timing and incidence of a composite of postoperative pulmonary complications. We hypothesized that ASA physical status, emergent case type, and procedure duration would be associated with pulmonary complications in high-risk children and that these complications would, in turn, be associated with a prolonged length of stay. Methods: Data from patients ≤ 6 years of age who were intubated for major abdominal surgery at the authors’ institution were collected from 1 January 2019 to 28 March 2022. The primary outcome was postoperative pulmonary complication, defined as the occurrence/use of reintubation, non-invasive positive pressure ventilation, high-flow nasal cannula, mask, or nasal cannula beyond phase 1 of recovery after anesthesia and within 7 postoperative days. The secondary outcome was hospital length of stay. We performed multivariable logistic regression with backward selection to identify independent predictors for postoperative pulmonary complications after adjusting for covariates. For hospital length of stay, a multivariate linear regression model was used after adjusting for covariates. Results: A total of 88 (26.1%) patients experienced 117 occurrences of postoperative oxygen dependence events, and 80 (90.9%) experienced this event in the first 48 h after surgery. The results of this model demonstrated independent associations between patients with an ASA class of IV (OR 9.86, 95% CI: 1.22–79, *p*-value = 0.03202) and longer operative time (OR: 1.05, 95% CI: 1.03–1.08, *p* = 0.00001) and postoperative pulmonary complication. On adjusted analysis, the occurrence of a postoperative pulmonary complication was associated with prolonged postoperative length of stay (adjusted geometric mean ratio of 1.39 (95% CI 1.10–1.75, *p* = 0.0062). Conclusions: Pediatric postoperative pulmonary complication remains a significant event for many patients and results in a prolonged length of stay. This study lays the groundwork for further investigations of interventions targeted at optimizing and monitoring at-risk individuals.

## 1. Introduction

Postoperative pulmonary complications (PPCs) are associated with increased in-hospital morbidity and mortality in pediatric populations [1,2]. PPCs manifest as a wide range of respiratory events including reintubation, prolonged mechanical ventilation, unplanned non-invasive ventilation, bronchospasm, pneumonia, and pulmonary edema. While PPCs are well-recognized in pediatric perioperative care, prior studies have primarily focused on respiratory failure requiring reintubation during limited postoperative time windows.

Consequently, the broader spectrum and time frame of PPCs in children remain poorly characterized. This lack of granularity limits our ability to detect earlier signs of PPCs and to design targeted interventions that capture patients at risk before the need for invasive respiratory support arises.

Children ≤ 6 years old are particularly vulnerable to PPCs because of their reduced pulmonary reserve, smaller airway anatomy, and increased susceptibility to perioperative respiratory insults. To address this gap, we examined a cohort of high-risk pediatric patients undergoing major abdominal surgery. Our primary objective was to evaluate the incidence and timing of a composite outcome of PPCs. We also assessed whether the occurrence of PPCs was associated with prolonged hospital length of stay (LOS).

We hypothesized that ASA physical status, emergent case type, and longer procedure duration would be associated with increased risk of PPCs, and that PPCs would in turn be independently associated with extended hospitalization.

## 2. Materials and Methods

### 2.1. Study Design

This study was approved as exempt by the Ann & Robert H. Lurie Children’s Hospital of Chicago Institutional Review Board (IRB 2021-4397; 5 February 2021). Because this was a retrospective cohort study, the requirement for informed consent was waived.

We included patients ≤ 6 years of age who underwent major abdominal surgery requiring endotracheal intubation at our institution between 1 January 2019 and 28 March 2022. The types of procedures classified as major abdominal surgery are listed in Supplemental Table S1. This subset of patients was selected based on prior evidence identifying them as being at the highest risk for postoperative pulmonary complications (PPCs) [2].

Exclusion criteria included patients ≥ 7 years old, those receiving preoperative supplemental oxygen, patients with a tracheostomy, and those with active COVID-19 infection at the time of surgery.

All anesthetics were delivered using Draeger Apollo anesthesia machines (Telford, PA, USA), which are maintained and serviced routinely according to institutional standards.

### 2.2. Data Collection

The following demographic and intraoperative variables were included: age, gender, height, actual body weight, American Society of Anesthesiologists physical status classification, case type (laparoscopic versus open procedure), operative time, case urgency, regional anesthesia adjunct, cyanotic heart disease, CNS tumor, history of cancer, impaired cognition/delayed, and a history of bronchopulmonary dysplasia. These variables were chosen based on prior studies showing independent association with postoperative respiratory failure [2].

### 2.3. Outcomes

The primary outcome was PPC, defined as the occurrence/use of reintubation, non-invasive positive pressure ventilation, high-flow nasal cannula, mask, or nasal cannula beyond phase 1 of recovery after anesthesia and within 7 postoperative days [2,3]. The secondary outcome was hospital length of stay (LOS).

### 2.4. Statistical Analysis

The characteristics of the sample were summarized using descriptive statistics. Continuous variables were reported as means ± standard deviations (SD), while categorical variables were expressed as counts (N) and corresponding percentages (%). Univariate logistic regression analysis was conducted to investigate factors associated with postoperative pulmonary complications. We performed multivariable logistic regression with backward selection to identify independent predictors for postoperative pulmonary complications after adjusting for covariates. For the outcome of hospital length of stay, a multivariate linear regression model was used after adjusting for covariates.

To ensure adequate statistical power for detecting clinically meaningful associations between risk factors and postoperative oxygen dependence (primary outcome, estimated prevalence 26%) in children aged ≤ 6 years undergoing major abdominal surgery, we conducted an a priori power analysis. Using logistic regression calculations performed in G*Power 3.1, we determined that detecting an odds ratio of 3.0 for a dichotomous risk factor (prevalence 20%) requires approximately 280 patients, assuming a two-sided α of 0.05 and 80% power. For operative duration as a continuous predictor, with an assumed standard deviation of 120 min, detecting a 5% increase in odds per 10 min increment necessitates about 330 patients. For the secondary outcome of hospital length of stay, detecting a 40% prolongation in patients with postoperative oxygen dependence, using log-transformed linear regression, also requires approximately 330 patients, based on a moderate effect size (Cohen’s d ≈ 0.67) and typical variability (coefficient of variation ≈ 0.5). This sample size ensures sufficient power to detect moderate-to-large effects for both primary and secondary outcomes. All statistical analyses were carried out using SAS software (version 9.4, SAS Institute Inc., Cary, NC, USA).

## 3. Results

### 3.1. Primary Outcome

**Figure 1:**
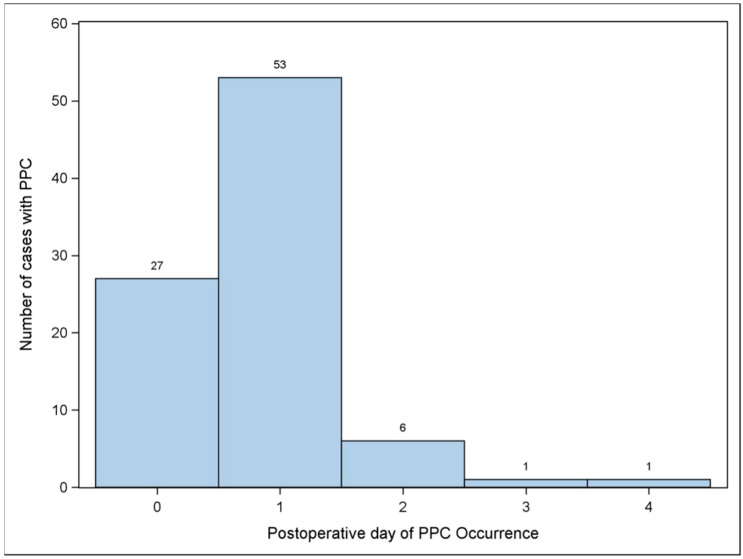
Total number of cases with PPC and the postoperative day of PPC occurrence.

A total of 375 pediatric surgery patients were identified from our dataset. Of these, 337 patients met our inclusion criteria and were included in the final analysis. Median age was 1.53 years (IQR 0.40, 3.00) with a median duration of anesthesia of 214 min (IQR 142, 321). A total of 88 (26.1%) patients experienced 117 occurrences of postoperative oxygen dependence events. 80 (90.9%) experience this event in the first 48 h after surgery. The modalities are summarized in Table 1. The number of cases with PPC and the day that PPC occurred are shown in Figure 1.

**Figure 2:**
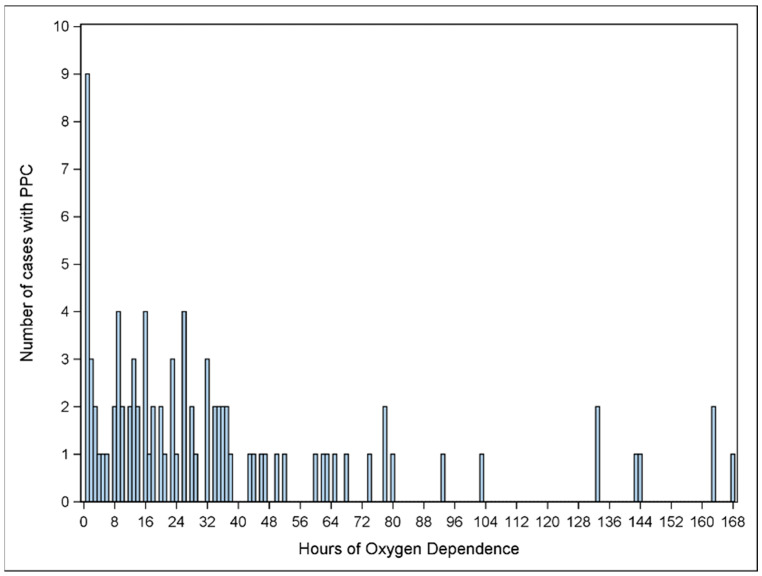
Total number of cases with PPC and the hours of oxygen dependence.

The results of the univariable analysis are shown in Table 2. Variables of statistically significant association, including ASA IV, operation time, regional anesthesia, impaired cognition, history of bronchopulmonary dysplasia, and fascial plane block, were input into the multivariable model. The results of this model, shown in Table 3, demonstrated independent associations between patients with an ASA class of IV (OR: 9.86, 95% CI: 1.22–79.9, *p*-value = 0.03202) and longer operative time (OR: 1.05, 95% CI: 1.03–1.08, *p*-value = 0.00001) with experiencing a postoperative pulmonary complication. Figure 2 shows the total number of cases with PPC and the hours of oxygen dependence.

### 3.2. Secondary Outcome

**Figure 3:**
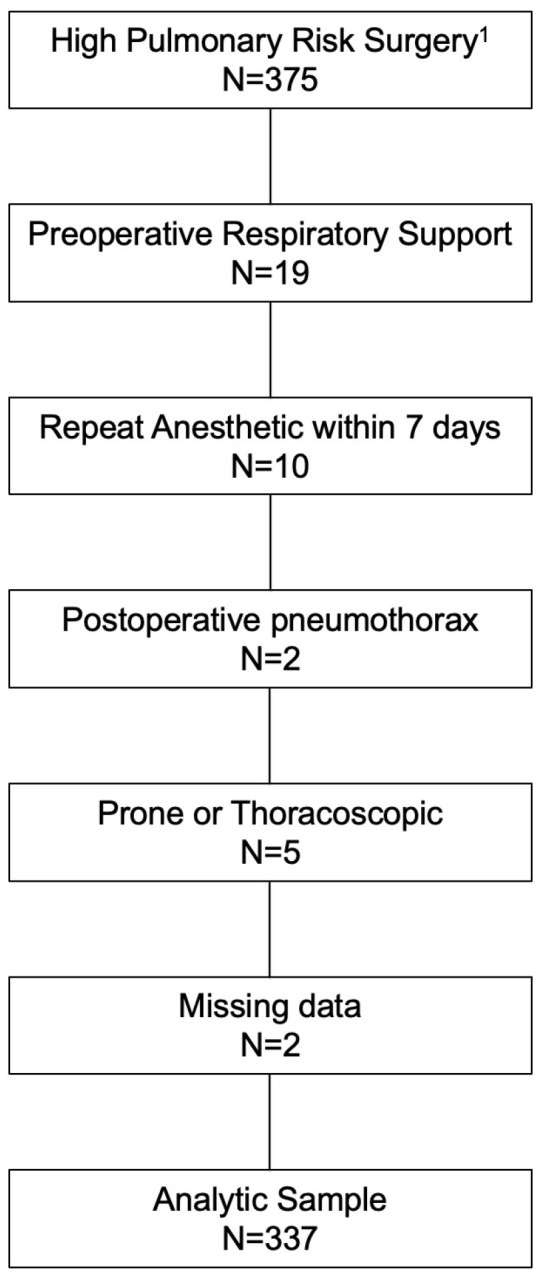
Attrition Flow Diagram. “^1^” is a reference from a source siting how defined those patients at high pulmonary risk for surgery.

On adjusted analysis, using the covariables of age, gender, and ASA status, the occurrence of a postoperative pulmonary complication was associated with prolonged postoperative length of stay (adjusted geometric mean ratio of 1.39 (95% CI 1.10–1.75, *p* = 0.0062) (Table 4). Figure 3 shows an attrition flow diagram.

## 4. Discussion

Approximately one quarter (26.1%, N = 88) of our patients experienced a postoperative pulmonary complication. Most often, this consisted of oxygen delivery via nasal cannula (67.5%). Less frequently, this involved more invasive modalities such as postoperative reintubation. There is limited evidence in the literature reporting on the postoperative course and frequency of postoperative pulmonary complications. Data has shown that intraoperative anesthetic management can impact pulmonary outcomes in adults even up to seven days after surgery [4]. Pediatric studies following patients with this degree of follow-up are scant. Ren and colleagues completed a retrospective review of postoperative pulmonary complications within seven postoperative days in 254 patients undergoing spine surgery at their institution. They found high tidal volumes to be associated with an increased incidence of postoperative pulmonary complications in older patients, and contrastingly, a decreased incidence in children < 3 years [5]. This study did not provide details on the occurrence of pulmonary complications by postoperative day. Further, this study examined a unique patient population in which cases are performed in the prone position, resulting in significant alteration of chest wall mechanics. Our cohort focused on patients we previously identified as being optimal for future interventional studies based on the high pulmonary complication rate and ease of identification for study enrollment [6]. Detailed characterization of the postoperative course in this high-risk cohort enables more informed planning for perioperative optimization, possible interventions as well as duration of postoperative outcome monitoring.

We found that the majority of postoperative pulmonary complications occurred on postoperative day 1 (90.9%, N = 80). This is in contrast to the adult literature, which shows a peak in desaturations on postoperative day 3 [7,8,9]. This gives an advantage to potential pediatric prospective trials in that the postoperative surveillance period could arguably be shortened. This suggests that interventions in the perioperative period made within the first 24 h could significantly impact a large proportion of patients. One study evaluating pediatric patients undergoing lung resection found that early respiratory therapy reduced the incidence of postoperative atelectasis [10]. Lee et al. recently showed that early high flow nasal cannula reduced the incidence of postoperative atelectasis as evidenced by lung ultrasound and PACU desaturation in children less than 3 years of age receiving general anesthesia for more than 2 h in duration [11]. Such an intervention might impact post-PACU pulmonary outcomes. A combination of preoperative and postoperative chest physiotherapy was found to be more effective at reducing postoperative pulmonary complications than postoperative physiotherapy alone in patients undergoing pediatric cardiac surgery [12]. A perioperative bundle in pediatrics directed at early interventions optimizing our higher-risk cohort warrants further study. Our study describes patient outcomes more holistically, in a way that affords more informed targeting in the planning of such an endeavor.

We found an ASA classification of 4 and increased operative time to be independently associated with a postoperative pulmonary complication. This is in line with previous studies looking at this population [2,13]. With just 10 patients included in our study with an ASA IV status and only 7 of these with a PPC, this lacks adequate power and warrants validation with further investigation of a larger patient subset. While perioperative management will always aim to optimize a patient’s comorbidities, this study provides another potential benefit of decreased pulmonary complications. A longer operative time can lead to the development of atelectasis and airway edema. For patients at increased pulmonary risk, consideration can be given to the utilization of technical advancements that shorten operative time and opting out of the bundling of multiple procedures under a single anesthetic. Interestingly, factors previously associated with pulmonary complications did not show an association in our study. Age and a history of cancer were not associated factors in our cohort [13,14,15]. Our exclusion criteria leave out those patients who are on supplemental oxygen preoperatively, as well as those who left the operating room intubated. Younger oncology patients undergoing major abdominal surgery may be more likely to fall into one of these categories and subsequently be eliminated from our study population. Impaired cognition/developmental delay, a known risk factor for pulmonary complications, did not reach statistical significance in our adjusted models. However, the confidence intervals suggest this may have been a factor but was underpowered in our study.

We found that postoperative pulmonary complication was associated with an increased postoperative length of stay. Increased hospital length of stay is a burden to each patient as well as the healthcare system due to increased costs. Oofuvong et al. attributed a twofold increase in LOS to perioperative respiratory events and a corresponding 30% higher excess hospital costs [1]. This does not account for the lost wages a family with a hospitalized child may suffer or other costs they may incur while being away from home. Intraoperative ventilatory management has been linked to length of stay in adults [16]. Medical optimization and targeted mitigation of risk may help to offset some of these costs [17].

Our study should be interpreted within the context of its limitations. It is a retrospective study from a single pediatric tertiary care center and therefore may not reflect practice patterns and patient populations at other institutions. Our study’s exclusion criteria, including age restrictions and preoperative conditions, may limit the generalizability of our findings to the broader population of pediatric patients undergoing major abdominal surgery.

Despite adjusting for various covariates, unmeasured confounding variables such as socioeconomic status, comorbidities, and perioperative management practices could influence the associations observed. Additionally, important variables such as parental smoking history, recent upper or lower respiratory tract infection, and detailed anesthetic management factors (e.g., airway techniques and use of specific anesthetic agents) were not uniformly available in the retrospective dataset and therefore could not be analyzed reliably. We agree these are important considerations and have explicitly acknowledged them here.

Furthermore, covariates such as intraoperative ventilation parameters, fluid balance, hypotension, or metabolic derangements—all of which are known to be associated with PPCs and LOS—were unavailable in our dataset. The absence of these variables may introduce residual confounding and limit the precision of our findings.

Our study lays the groundwork for further investigations of interventions targeted at optimizing and monitoring at-risk individuals. With this investigation, the need for validation with a future large-scale multicenter randomized controlled trial is highlighted. PPCs in high-risk pediatric patients occur early, are associated with prolonged hospitalization, and represent an important target for intervention. By clarifying their timing and risk factors, our study supports the development of early perioperative strategies and evidence-based care bundles aimed at improving outcomes and reducing healthcare burden.

## Figures and Tables

**Table 1:** Types of postoperative oxygen dependence.

Delivery Type	N (%)
Nasal Cannula	79 (67.5)
Simple Mask	3 (2.6)
Blow By	14 (12.0)
High Flow Nasal Cannula	9 (7.7)
Non-Rebreather	4 (3.4)
Non-Invasive Positive Pressure Ventilation	2 (1.7)
Endotracheal Tube	6 (5.1)

**Table 2:** Univariable analysis of patients with and without postoperative oxygen dependence.

	Mean ± SD/N (%)			
Variable	No Postoperative Oxygen Dependence (N = 248)	Postoperative Oxygen Dependence (N = 89)	Unadjusted OR (95% CI)	*p*-Value
Age (y)	1.93 ± 1.82	2.32 ± 1.99	1.11 (0.98, 1.26)	0.0931
Sex				
Male	118 (47.58)	49 (55.06)	1.35 (0.83, 2.19)	0.2270
Female	130 (52.42)	40 (44.94)	1	REF
Height (cm)	92.92 ± 19.48	95.62 ± 17.16	1.01 (0.99, 1.02)	0.2473
Actual Body Weight (kg)	11.31 ± 6.69	11.05 ± 5.57	0.99 (0.96, 1.03)	0.7401
ASA Physical Status				
I	16 (6.45)	2 (2.25)	1	REF
II	97 (39.11)	18 (20.22)	1.48 (0.31, 7.02)	0.6184
III	132 (53.23)	62 (69.66)	3.76 (0.84, 16.84)	0.08384
IV	3 (1.21)	7 (7.87)	18.66 (2.53, 137.5)	0.00409
Case type				
Laparoscopic	41 (16.53)	9 (10.11)	1	REF
Open	191 (77.02)	77 (86.52)	1.84 (0.85, 3.96)	0.12104
Operative time (min)	219.71 ± 111.64	323.35 ± 154.71	1.06 (1.04, 1.08)	<0.0001
Emergency	42 (16.94)	10 (11.24)	0.62 (0.30, 1.30)	0.2048
Regional Anesthesia	94 (37.90)	45 (50.56)	1.68 (1.03, 2.73)	0.0383
Cyanotic Heart Disease	2 (0.81)	3 (3.37)	4.29 (0.71, 26.11)	0.1140
CNS Tumor	3 (1.21)	0 (0)	0.39 (0.01, 12.10)	0.5928
Impaired Cognition/Delayed	26 (10.48)	18 (20.22)	2.17 (1.12, 4.18)	0.0214
History of Cancer	50 (20.16)	12 (13.48)	0.61 (0.31, 1.21)	0.1616
History of Bronchopulmonary Dysplasia	1 (0.40)	4 (4.49)	11.62 (1.28, 105.35)	0.0292
Neuraxial				
None	153 (61.69)	44 (49.44)	1	REF
Fascial plane block	39 (15.73)	27 (30.34)	2.41 (1.33, 4.36)	0.00376
Neuraxial	56 (22.58)	18 (20.22)	1.12 (0.60, 2.09)	0.7284

OR, odds ratio; ASA, American Society of Anesthesiologists; CNS, central nervous system.

**Table 3:** Independent associations with postoperative oxygen dependence, multivariable analysis.

	Mean ± SD/N (%)			
Variable	No Postoperative Oxygen Dependence (N = 248)	Postoperative Oxygen Dependence (N = 89)	Adjusted OR (95% CI)	*p*-Value
Age (y)	1.93 ± 1.82	2.32 ± 1.99	1.01 (0.87, 1.18)	0.85092
ASA Physical Status				
I	16 (6.45)	2 (2.25)	1	REF
II	97 (39.11)	18 (20.22)	0.95 (0.19, 4.69)	0.95167
III	132 (53.23)	62 (69.66)	1.61 (0.34, 7.65)	0.54603
IV	3 (1.21)	7 (7.87)	9.86 (1.22, 79.9)	0.03202
Operative time (min)	219.71 ± 111.64	323.35 ± 154.71	1.05 (1.03, 1.08)	0.00001
Impaired Cognition/Delayed	26 (10.48)	18 (20.22)	1.84 (0.88, 3.86)	0.10546
History of Bronchopulmonary Dysplasia	1 (0.40)	4 (4.49)	4.74 (0.47, 47.68)	0.1869
Neuraxial				
None	153 (61.69)	44 (49.44)	1	REF
Fascial plane block	39 (15.73)	27 (30.34)	1.11 (0.53, 2.29)	0.78678
Neuraxial	56 (22.58)	18 (20.22)	0.69 (0.34, 1.4)	0.30278

OR, odds ratio; ASA, American Society of Anesthesiologists.

**Table 4:** Association between the Postoperative Oxygen Dependence groups and outcome variables.

Outcomes	Mean ± SD			Unadjusted Model		Adjusted Model	
	Total Sample	Group		Geometric Mean Ratio (95% CI)	*p* value	Geometric Mean Ratio (95% CI)	*p* value
	N = 337	No Postoperative Oxygen Dependence	Postoperative Oxygen Dependence				
		N = 248 (73.59%)	N = 89 (26.41%)				
Postoperative Length of Stay	11.51 ± 15.44	9.91 ± 14.65	15.98 ± 16.73	1.95 (1.55–2.45)	<0.0001	1.39 (1.10–1.75)	0.0062

SD = standard deviation; CI = confidence interval; No Postoperative Oxygen Dependence is the referent group. Length of Stay and Postoperative Length of Stay are not normally distributed, and they are log-transformed and used in the linear regression model.

## Data Availability

The original contributions presented in this study are included in the article/Supplementary Material. Further inquiries can be directed to the corresponding author.

## Supplementary Materials

The following supporting information can be downloaded at: https://www.mdpi.com/article/10.3390/children12101403/s1, Table S1: Surgical procedures included in analysis.

## Abbreviations

The following abbreviations are used in this manuscript:

ASA	American Society of Anesthesiologists
CI	confidence interval
IRB	institutional review board
LOS	length of stay
OR	operating room
PPC	postoperative pulmonary complication

## References

[1] Oofuvong M., Geater A.F., Chongsuvivatwong V., Chanchayanon T., Sriyanaluk B., Saefung B., Nuanjun K. (2015). Excess costs and length of hospital stay attributable to perioperative respiratory events in children. Anesth. Analg..

[2] Cheon E.C., Palac H.L., Paik K.H., Hajduk J., De Oliveira G.S., Jagannathan N., Suresh S. (2016). Unplanned, Postoperative Intubation in Pediatric Surgical Patients: Development and Validation of a Multivariable Prediction Model. Anesthesiology.

[3] Karalapillai D., Weinberg L., Peyton P., Ellard L., Hu R., Pearce B., Tan C.O., Story D., O’Donnell M., Hamilton P. (2020). Effect of Intraoperative Low Tidal Volume vs Conventional Tidal Volume on Postoperative Pulmonary Complications in Patients Undergoing Major Surgery: A Randomized Clinical Trial. JAMA.

[4] Futier E., Constantin J.-M., Paugam-Burtz C., Pascal J., Eurin M., Neuschwander A., Marret E., Beaussier M., Gutton C., Lefrant J.-Y. (2013). A trial of intraoperative low-tidal-volume ventilation in abdominal surgery. N. Engl. J. Med..

[5] Ren Y., Liu J., Nie X., Liu L., Fu W., Zhao X., Zheng T., Xu Z., Cai J., Wang F. (2020). Association of tidal volume during mechanical ventilation with postoperative pulmonary complications in pediatric patients undergoing major scoliosis surgery. Paediatr. Anaesth..

[6] Cheon E.C., Ballard H.A., Burjek N.E., Phillips M., Robles A., Raval M.V. (2022). Identifying a pediatric cohort to prospectively evaluate ventilation strategies to mitigate postoperative pulmonary complications. Paediatr. Anaesth..

[7] Rosenberg J., Ullstad T., Larsen P.N., Moesgaard F., Kehlet H. (1990). Continuous assessment of oxygen saturation and subcutaneous oxygen tension after abdominal operations. Acta Chir. Scand..

[8] Rosenberg J., Ullstad T., Rasmussen J., Hjorne F.P., Poulsen N.J., Goldman M.D. (1994). Time course of postoperative hypoxaemia. Eur. J. Surg..

[9] Rosenberg J., Wildschiodtz G., Pedersen M.H., von Jessen F., Kehlet H. (1994). Late postoperative nocturnal episodic hypoxaemia and associated sleep pattern. Br. J. Anaesth..

[10] Kaminski P.N., Forgiarini L.A., Andrade C.F. (2013). Early respiratory therapy reduces postoperative atelectasis in children undergoing lung resection. Respir. Care.

[11] Lee J.H., Ji S.H., Jang Y.E., Kim E.H., Kim J.T., Kim H.S. (2021). Application of a High-Flow Nasal Cannula for Prevention of Postextubation Atelectasis in Children Undergoing Surgery: A Randomized Controlled Trial. Anesth. Analg..

[12] Felcar J.M., Guitti J.C., Marson A.C., Cardoso J.R. (2008). Preoperative physiotherapy in prevention of pulmonary complications in pediatric cardiac surgery. Rev. Bras. Cir. Cardiovasc..

[13] Eisler L.D., Hua M., Li G., Sun L.S., Kim M. (2019). A Multivariable Model Predictive of Unplanned Postoperative Intubation in Infant Surgical Patients. Anesth. Analg..

[14] Subramanyam R., Yeramaneni S., Hossain M.M., Anneken A.M., Varughese A.M. (2016). Perioperative Respiratory Adverse Events in Pediatric Ambulatory Anesthesia: Development and Validation of a Risk Prediction Tool. Anesth. Analg..

[15] Katz S.L., Monsour A., Barrowman N., Hoey L., Bromwich M., Momoli F., Chan T., Goldberg R., Patel A., Yin L. (2020). Predictors of postoperative respiratory complications in children undergoing adenotonsillectomy. J. Clin. Sleep. Med..

[16] Guay J., Ochroch E.A., Kopp S. (2018). Intraoperative use of low volume ventilation to decrease postoperative mortality, mechanical ventilation, lengths of stay and lung injury in adults without acute lung injury. Cochrane Database Syst Rev..

[17] Ludbrook G.L. (2022). The Hidden Pandemic: The Cost of Postoperative Complications. Curr. Anesthesiol. Rep..

