# Supplementary material for: Risk Factors for Postoperative Pulmonary Compromise in a Pediatric Population: A Retrospective Review of a Single Institution Cohort
Source: Children (Basel). 2025 Oct 17;12(10):1403. doi: 10.3390/children12101403 (PMC12564788; doi:10.3390/children12101403)
Supplement: Supplementary file 1 [file children-12-01403-s001.zip › children-3818010-supplementary.pdf]

Table S1. Surgical procedures included in analysis		
**Procedure Name**	**N**	**%**
Laparoscopy, surgical; gastrostomy, without construction of gastric tube (eg, Stamm procedure)	140	8.8
Laparoscopy, surgical, esophagogastric fundoplasty (eg, Nissen, Toupet procedures)	96	6.0
Closure of enterostomy, large or small intestine; with resection and anastomosis other than colorectal	70	4.4
Esophagoplasty for congenital defect, thoracic approach; with repair of tracheoesophageal fistula	68	4.3
Esophagogastric fundoplasty partial or complete; laparotomy	32	2.0
Closure of enterostomy, large or small intestine	26	1.6
Enterectomy, resection of small intestine; single resection and anastomosis	24	1.5
Correction of malrotation by lysis of duodenal bands and/or reduction of midgut volvulus (eg, Ladd procedure)	19	1.2
Gastrostomy, open; without construction of gastric tube (eg, Stamm procedure)	19	1.2
Enteroenterostomy, anastomosis of intestine, with or without cutaneous enterostomy	16	1.0
